# A behavioural intervention for young adults with dental caries, using acceptance and commitment therapy (ACT): treatment manual and case illustration

**DOI:** 10.1186/s12903-020-01213-4

**Published:** 2020-08-26

**Authors:** Helene Werner, Celia Young, Magnus Hakeberg, Ulla Wide

**Affiliations:** 1 Department of Behavioral and Community Dentistry, Institute of Odontology, The Sahlgrenska Academy, University of Gothenburg, P.O. Box 450, SE-40530 Gothenburg, Sweden; 2 Psykologpartners AB, Gothenburg, Sweden

**Keywords:** Acceptance and commitment therapy (ACT), Behavioural intervention, Treatment manual, Dental caries, Oral health

## Abstract

**Background:**

There is a need for effective behavioural interventions in dentistry. This paper presents an innovative behavioural intervention for young adults with dental caries, in an interdisciplinary collaboration between dental personnel and licensed psychologist in general dental care. The intervention has been evaluated in an RCT, with positive effects on oral health behaviour.

**Method:**

The intervention, Acceptance and Commitment Therapy (ACT), a recent form of cognitive behaviour therapy (CBT), was adapted to young adult patients (18–25 years of age) with high dental caries activity.

**Results:**

The intervention included two individual sessions, provided by a clinical psychologist at general dental clinics. The rationale for selecting ACT as the theory base is presented, together with the treatment manual and a case illustration.

**Conclusions:**

ACT may be a promising alternative for behavioural interventions in dentistry for patients with oral diseases, specifically dental caries. Interprofessional collaboration between psychologists and dental personnel opens up for new possibilities to help and treat patients with various health issues in public dental care.

**Trial registration:**

TRN ISRCTN15009620www.isrctn.com, retrospectively registered 14/03/2018.

## Background

Dental caries is a major public health issue, affecting 60–90% of adolescents and nearly all adults worldwide [1, 2]. Early stages of the disease may go unnoticed, whereas advanced states with pulp and jaw infections often cause severe pain, impaired oral function, social limitations and impaired quality of life [1]. In addition, the disease is associated with school absenteeism and reduced work activity as well as extensive treatment costs. Importantly, dental caries is strongly related to health behaviour (sugar consumption, oral hygiene, use of fluoride and dental care attendance).

Traditionally, interventions for patients with dental caries include prevention, oral health education and restorative treatment. Oral health education increases patients’ knowledge of oral health, but has limited effects on oral health-related behaviour and other clinically relevant outcomes [3]. High-quality studies on theory-driven behaviour interventions have been called for [4, 5].

Health behaviour science provides several well-established theories of behaviour and behavioural change. The theory of planned behaviour and the transtheoretical model of behavioural change, for instance, have influenced interventions in dental care [6]. In a systematic Cochrane review [7], including four randomised controlled trials (RCTs), there was some evidence that behavioural interventions could improve oral hygiene in periodontal patients. In another systematic review [8] including ten RCTs, behavioural interventions had a small but statistically significant positive effect on interdental cleaning and toothbrushing in patients with periodontitis. However, the certainty of evidence in these systematic reviews was low, and there were few studies evaluating behavioural interventions for adolescents and young adults with dental caries as well as other oral diseases.

Acceptance and Commitment Therapy (ACT) [9] is a recent form of cognitive behaviour therapy (CBT), based on relational frame theory [10]. In several systematic reviews [11–15], ACT has shown small to moderate mean effect sizes for psychological (e.g., depression and anxiety) and somatic (e.g., chronic pain and tinnitus) diseases and disorders, when compared to control conditions (i.e., treatment as usual, waiting list and placebo). However, to our knowledge, ACT has not been tested in dental care. Thus, ACT may be a promising candidate for theory-based interventions in dentistry. ACT was not developed for a particular disease, but is applicable where behavioural change is needed [16], and patients with dental caries may need to make several behaviour changes (sugar consumption, oral hygiene, exposure to fluoride and dental care attendance) [6]. Also, there are brief ACT formats for primary care settings [17], and Powers et al. [13] found that short versions of ACT (e.g., one-time workshops) had similar effect sizes as longer-term therapy. ACT could be especially useful when the goal, as in dentistry, is to promote health, and not just treat a pathology [18]. The processes of change in ACT are different from those in educational approaches [19, 20], such as oral health education with limited effects on oral health [3, 8]. We propose that an ACT-consistent focus on values may be especially useful in clarifying how oral health behaviour can be relevant to valued life directions and that this will lead to behavioural change.

Thus, we developed a two-session manual-based ACT behavioural intervention for young adult patients with dental caries. The intervention was evaluated in an RCT where positive effects on oral health behaviour were found, and significantly more in the ACT intervention group than in the control group [21]. The large majority of patients completed the study, which is promising with regard to treatment acceptability, as this age group is known for the highest therapy dropout rates [22]. Moreover, the intervention tested interdisciplinary collaboration between dental personnel and psychologists in general dental care, previously proven effective for patients with dental phobia and implemented in specialised dental care [23] but, as far as we know, new to general dental care clinics.

## Methods

### Aim

The aim of the present paper was to present the rationale and the treatment manual (including a case illustration) for a brief behavioural intervention for young adults with dental caries. The intervention was an adapted form of ACT, provided by a clinical psychologist, for use in general dental clinics.

### ACT concept of change and its relevance for young adults with dental caries

The overall aim in ACT is increased psychological flexibility, which enables the individual to maintain functional behaviours and to change dysfunctional ones, in order to live in accordance with chosen life values [12]. According to ACT, there are six separate, but interrelated, concepts of change, which work both as treatment processes and skills to be practiced by the patient [12, 24]. The relevance of these concepts for young adults with dental caries is presented below.

*Acceptance* includes a willingness to experience and explore memories, thoughts, feelings and bodily sensations, also when they are uncomfortable [16]. The opposite is experiential avoidance [25]. Patients with dental caries may eat sweets, for example, in order to control stress or tiredness, avoid flossing because of the risk of bleeding, or cancel an appointment with the dentist due to anxiety. Through acceptance, the patient can learn to look at these patterns in a non-judgemental way, making it easier to change their habits and behaviour [12].

*Mindfulness* exercises aim to increase the awareness of the present moment [16]. Caries-active patients may ruminate about the origins of their current oral health and get stuck in thinking about their parents getting them addicted to sugar or being poor role models. They may also view the future rather than the present as the time for change. In therapy, mindfulness exercises are especially useful when there is avoidant behaviour (e.g., superficial talk, humorous avoidant behaviour, or constant changing of the subject) [26].

*Defusion* is a process and an ability to recognise thoughts and mental events for what they are, rather than as true representations of reality [24]. A painful visit to the dentist, for instance, may lead to the avoidance of dental care. Alternative strategies or the possibility that new experiences could be different may be forgotten. Defusion is also needed when thoughts and words are taken at face value, leading to dysfunctional behaviour [26]. A patient with dental caries may say: “I can’t brush my teeth in the morning because I don’t have time, and in the evening I am too tired.” Such thoughts may seem true and real for the patient, and the brushing frequency appears difficult to change. However, objectively, time is relative, habits are changeable, and tiredness is a possible feeling to experience while brushing one’s teeth.

*Self as a Context* makes it possible to see oneself, situations and obstacles from a broader perspective [26]. Each patient has his/her own unique experiences of dental care and oral hygiene. They also have expectations, habits and stories about who they are. Patients may say: “I am lazy, so I don’t brush my teeth,” “I am addicted to sugar, so I eat sweets every day,” and/or “I need 10 cigarettes per day.” These fused self-stories may influence oral health negatively and prevent constructive behaviour.

*Values:* According to the theory behind ACT, people are often out of touch with their values in life and their behaviour is guided by other factors [12]. We find it reasonable to assume that most humans value self-care, if for no other reason than to be able to focus on other valued areas of life. However, many people may not ponder their values concerning oral health and may not be aware of any connection between such self-care and other parts of their life. In ACT, values are seen as a key factor for change and as long-term guidance for meaningful actions in life [16]. Oral and general health-related values may help patients change their oral health-related behaviour and sustain such change over time.

*Committed Action* includes the ability and willingness to engage in valued behaviour [12]. Challenges to change may occur in therapy. For example, when mapping obstacles to smoking cessation, the patient may feel overwhelmed, present rigid stories about themselves and display avoidant behaviour in the room. The ACT strategies described above could then be helpful. Committed action is put to the test by homework between sessions; for example, a patient may notice the urge to have another cigarette, but choose not to for the sake of oral health-related values. Committed action is also addressed through efforts to prevent and deal with potential and/or actual setbacks in therapy.

### Development of the ACT intervention

The ACT intervention was developed and adapted for young adults with dental caries by the authors HW, CY and UW, all clinical psychologists with CBT and ACT competence, in collaboration with a specialist in dentistry (MH). The intervention was documented in a manual specifying the included ACT exercises. The intervention was tested in several patients, resulting in the selection of the main exercises, including the Bull’s Eye [27] and a defusion exercise [28], with only minor adaptations to suit the study format. In addition, we developed an ACT-consistent exercise called “Mindful oral health”, described below. HW who is a licensed psychologist with advanced training in ACT plus working with CBT in specialised dental care, delivered the intervention, and CY supervised HW during the whole study period.

## Results

### Treatment manual

The ACT treatment manual for young patients with dental caries is outlined below. The patient receives two individual ACT sessions (45 min each), two to three weeks apart, delivered by a clinical psychologist with competence in CBT and ACT at a general dental clinic. To increase treatment adherence, work sheets are used for the sessions. For a treatment overview, see Table 1.

**Table 1:** Treatment overview

Session 1	Session 2
Introduction Brief interview Mindful oral health Focused questions Case conceptualisation Clarification of values Defusion exercises Plan for behavioural change Concluding comments	Follow-up Mindful oral health Clarification of values Defusion exercises Plan for behavioural change Conclusion

#### Session 1

##### Introduction

The psychologist welcomes the patient and asks about his/her expectations and hopes for the session. If the patient seems ambivalent or hesitant, potential underlying values guiding the patient’s choice to participate in the intervention in the first place are investigated. The aim (1) and rationale (2) of the intervention are then presented: 1. “This is a health counselling session, with the aim to explore possible improvements of your oral health.” 2. “I will first ask you some questions about your life situation and then about your oral health, after which we will discuss a plan for you to improve your oral health. How does that sound? We have about 45 minutes today and another session in two weeks.”

##### Brief interview

The patients are asked questions about their life (in line with the “Love, work, play” section in Strosahl et al. [17]), general health (e.g., medication, tobacco, alcohol and drug use), and oral health (e.g., food and drinking habits, toothbrushing, flossing and use of fluoride). They also get to describe their own oral health and rate how much of an issue it is today (on a scale from one to ten, ten meaning that their oral health is experienced as a great problem).

##### Mindful oral health

The patients are asked to notice the current status of their oral health. The psychologist assists the patient with instructions, such as, “If you feel your gum and teeth, how do they feel today?”. The exercise aims to increase the awareness of oral health but can also give an indication of the patient’s openness and engagement in the treatment.

##### Focused questions

At this stage, behaviour of importance to the patient’s oral health is summarised and the patient is asked if there is any behaviour that he/she is willing to try to change to improve their oral health. Clinically relevant information is then gathered through the focused questions in a “Focused interview” [17], including: “What have you tried?”; “How has it worked?”, and “What has it cost you (or what might be the cost in the future)?”

##### Case conceptualisation

During the work described above, a behaviour analysis is performed [29] for a deeper understanding of the chosen behaviour to be changed. The psychologist also completes the “Four Square Tool” [17] and rates the patient’s levels of openness, awareness and engagement (on a scale from one to ten, where ten is the highest) to generate a hypothesis about effective ACT interventions for this particular patient.

##### Clarification of values

The patient is encouraged to think about what oral health he/she desires, how it might feel, why it is important and what the patient would be able to do. Patients may wish for whole and white teeth to eat and smile with, and to feel fresh so that they can kiss their loved ones. A modified version of the Bull’s Eye Value Survey [27] is also used, where the patients write down their oral health-related values, and put an “X” on a drawn dartboard to signify how close to, or far away from, their values (at the centre of the dartboard) they are today. They are also asked to write down their obstacles to change (e.g., urge for sweets, the thought “I need something sweet”, sadness) and rate their intensity on a scale from one to ten (where one is low).

##### Defusion exercises

The psychologist actively addresses fused material that could be of importance for the patient’s oral health behaviour. The psychologist describes and labels thoughts as thoughts and asks perspective-taking questions.

##### Plan for behavioural change

The patient specifies by writing a plan for behavioural change (e.g., what to do, when to do it and how to do it) and helpful strategies when faced with obstacles. The psychologist may add suggestions, such as functional strategies that the patient has mentioned or shown in terms of ACT-relevant skills. The patients are also asked to rate how likely it is that they will comply with the behavioural plan on a scale from one to ten. If the patient rates below six and there is time left, the plan is revised, otherwise the work continues in Session 2. The patients also get a copy of the Bull’s Eye to take home.

#### Session 2

##### Follow-up

The patient is asked how the prior session was experienced and to describe how the plan for behavioural change has worked. Additional strategies, solutions and obstacles are noted. Setbacks are separated from relapses, and value-driven actions highlighted as more important than reaching goals. The patient is then asked to rate how much of an issue their oral health is today on a scale from one to ten (as in Session 1).

##### Mindful oral health

The patient is asked to perform the same mindful oral health exercise as in Session 1.

##### Clarification of values

The psychologist provides a copy of the patients’ Bull’s Eye Value Survey from Session 1 and a new one. The patient is asked to put a new “X” on the second dartboard, representing how close to their values they are at this point. The patients are asked to reflect on how they feel, if they are satisfied with where the “X” is now or would like to discuss possible actions to reach the Bull’s Eye. The patient writes down what has worked since the last occasion (i.e., what to continue with), possible obstacles in the year to come and helpful strategies to handle them.

##### Defusion exercises

In addition to the strategies used in session 1, the exercise found in Harris [28] is used. The patient is asked to write down recurring obstacles to toothbrushing, such as the thought, “I’m too tired,” on a post-it sticker, and experiment with the physical distance to it and its influence on the patient’s possibility to view and reach the Bull’s Eye. Mindfulness and acceptance often complement this exercise.

##### Plan for behavioural change

In this session, a new written behavioural change plan is formulated. The psychologist helps the patient to formulate realistic and specific plans for change and normalises and helps the patient handle possible setbacks. The patient rates the likelihood of each suggested behavioural change being carried out on a scale from one to ten. If the rating is below six, additional work is considered. The patient gets a copy of the new Bull’s Eye to take home. The psychologist encourages the patient to continue to attend the dental clinic for examinations, health promotion and treatments, and to take an active role in the treatment planning.

### Case illustration

This is a hypothetical case illustration, constructed using material from several individuals.

#### Session 1

The (fictional) patient Eva (E) is 22 years old and works at a café. According to the focused interview, E lives with her parents during the week and with her boyfriend at weekends. She brushes her teeth every morning, but rarely in the evening. E drinks a can (33 cl) of soft drink on a daily basis, and about one litre on weekends. E is overweight, says she often feels stressed and smokes half a pack of cigarettes every day. E has had a number of restorative dental treatments due to dental caries. During the mindful oral health exercise, E notices her latest two fillings, and the taste of a snack she ate just before the session. She rates her oral health as an issue as a four on a ten-point scale (ten being the maximum). When her oral health-related behaviours are summarised, E says she would like help to drink less soft drink.

During the functional behavioural assessment E describes that she started drinking soda on a daily basis when she got a job at a café. She stopped drinking it for a couple of months when she was out of work, but started again when she got another job at a café with easy access to it. “I usually open a can after an hour at work and sip on it until lunch, and on weekends I share a two-litre bottle with my boyfriend.” When asked the focused questions, E says that she drinks soft drinks because she loves the taste and wants to stay alert. She knows it only works momentarily, because she is often thirsty or tired again after a while. When asked about costs, E says that it is not that expensive. The psychologist rates E as being fairly open, but not in contact with her emotions at this stage of therapy. When asked about other costs of soft drink consumption, E says diabetes. The psychologist notices how E clenches her teeth and looks away. The psychologist asks if E notices what is happening inside her at that moment. E says: “Nothing”, but then “Anxiety”. The psychologist asks where in the body and E points at her chest. The psychologist normalises this and credits E for being in touch with her feelings. E says that she has seen the consequences of diabetes. With a bit of encouragement, she describes those consequences. Afterwards E sighs, and says she really does not want that for herself.

The session naturally shifts focus to values. When E is asked about what oral health she desires instead she first replies: “Whole and white teeth”. When asked why that is important (for more genuine values), E answers that it is a sign of taking care of oneself. And when asked to elaborate, E says that she wants to be healthy. The psychologist asks what E would be able to do then, whereby E answers with emphasis: “Have children!” The psychologist: “It sounds like that is truly important for you?” “Yes, I have always dreamt of having children”. E then summarises her values in writing: “It is important for me to have whole and white teeth, to take care of myself, remain free from illness and be able to have children”. E puts an “X” halfway to/from the centre of the Bull’s Eye dartboard. E writes “stress” and “the urge for soft drink” as her main obstacles to change, and rates them as a seven on a ten-point scale. She writes that she could try to stop drinking soft drink and drink water instead. After considering what else might be helpful, she adds: “Not carrying coins with me, and tell my family about my plan for better teeth”. When asked how likely it is that she won’t drink soft drink the next two weeks she says eight on a ten-point scale.

#### Session 2

E immediately says: “I have stopped drinking soft drinks! Can you imagine?” According to E, it was a great challenge the first week, with tiredness and an urge for soft drink, but she had stuck to the plan and the urge wore off. E says that she has thought a lot about her teeth, and that she told her family and boyfriend, who suggested brushing together in the evenings, which has worked well. E says that she has also started to pay off a loan using the money she saves on not drinking soft drink. She seems happy and says she also feels proud. E rates her current oral health as a three as an issue on a ten-point scale, and puts her “X” one step closer to the Bull’s Eye (the middle of the dartboard). When doing the oral mindful exercise, E notices freshness in her mouth after brushing before the session; however, E says that she has thought about another habit detrimental to her health: chocolate drinks. E says she drinks eight glasses per day, and has done so since she was a teenager. Similar factors contribute to these behaviours.

The psychologist introduces the defusion exercise. E writes, “tastes good”, on a post-it sticker. During the exercise, E identifies more obstacles, such as tiredness, stress and the thoughts, “I need chocolate”, “I must have chocolate”. When asked to show how persuasive these thoughts may be, E puts the post-it stickers on her face. When asked how well she sees the Bull’s Eye, she says: “I don’t see it at all!” E laughs and speeds up. The psychologist shares noticing that E laughs, but also asks how she feels inside right now. E pauses and says: “Actually, I feel that anxiety in my chest again”. The psychologist helps E stay with that emotion. E slows down and says: “All this (points at the post-it sticker) feels overwhelming sometimes, but at the same time, I know I can change. I mean I stopped drinking soft drink! I had all these thoughts but I didn’t listen to them.” The psychologist praises E for noticing, and recommends her to write that down. E writes: “I can notice but not act on my thoughts or urges for soft drinks and chocolate”. E specifies a plan for how to keep brushing twice a day, for not drinking soft drink and for giving up chocolate drinks. After information about potential setbacks, E rates the likelihood of her following her plan as a ten out of ten. E still smokes, but seems more willing and committed to seizing opportunities for a healthier life.

## Discussion

In this paper, we have presented the manual for a brief behavioural intervention for young adults with dental caries. The intervention was a cognitive behaviour therapy method called Acceptance and Commitment Therapy (ACT) delivered by a clinical psychologist at a general dental care clinic.

ACT has previously shown promising results for different patient groups [11, 12, 14, 15, 30, 31], but is, to our knowledge, new for patients with an oral disease such as caries. We have suggested how the core concepts of change in ACT may work for patients with dental caries; for example, through acceptance of contributing factors to poor oral health and openness to alternative strategies, mindful oral health and awareness of influential emotions regarding dental care, sugar habits, tobacco use and oral hygiene, defusion of rigid self-stories leading to unhealthy habits, and, perhaps most importantly, clarified values associated with oral health, whereby committed action for a healthier life might be possible. Interestingly, there are contrasting differences between ACT and traditional patient education in dentistry. While traditional patient education is rather directive, ACT is person-centred. The patient chooses what behaviour to change, and the psychologist helps the patient to get in contact with values that could guide behaviour change, and to increase his/her skills to handle hinders. Thus this is a health promotive intervention, as patients improve their capacity for healthier behaviour in general.

Previous studies on behavioural interventions in dentistry have been criticised for methodological weaknesses, such as inadequate sample size [7, 8]. ACT studies have been criticised especially for the choice of study design [15]. The present intervention has been evaluated in an RCT, showing high acceptability and positive effects on oral health behaviour [21].

In addition to the request for more theory-based behavioural interventions [32], the need to publish and make detailed treatment manuals accessible has been acknowledged [8]. Access to treatment manuals makes critical evaluation of the interventions possible, and is also necessary for replications and for the further development of clinical research. It has been argued that for many interventions presented as theory-based, the publications provide limited information about how the intervention is actually related to the theory [33]. The present paper includes a detailed rationale for how ACT concepts, processes and exercises are related to the target population and used in the specific adapted intervention.

There is growing interest in developing evidence-based interventions for behaviour change within dentistry. There are several areas in clinical dentistry where behaviour change plays an important part for treatment, prevention and health promotion, and various methods may be needed to address different problems [6, 34]. ACT has the potential to be effective for many patients in dentistry, not only patients with dental caries, but also patients with periodontitis, erosion problems, pain and tendencies to vomit or gag. However, more research is needed to evaluate the efficacy and effectiveness of ACT before it can be implemented in dental care. Results on more outcomes and long-term effects are analysed at present.

Common questions about behavioural interventions concern the dose, the use of an individual or group setting, and the provider of the intervention. The format of a brief intervention was selected to attract patients with limited time and varying motivation for treatment; i.e., young adults, and to make it possible to treat more patients in a cost-effective way. However, a booster session performed either at the dental clinic or by phone might enhance the effect. Another important issue is which profession is best suited to provide behavioural interventions. A systematic review reported that different professions were used (such as dentist, dental hygienist, psychologist); however, the difference in effect between different providers could not be evaluated [8]. In the studies (*n* = 26) concerning pain, tinnitus, depression and anxiety, included in the systematic review by Öst [15], the clear majority of therapists who provided ACT were psychologists, psychotherapists or psychology students (at the master degree level). We strongly believe that the present ACT intervention requires a clinical psychologist with CBT and ACT competence. We are convinced that this is necessary for performing the functional analysis of behaviour and using the ACT interventions correctly, especially in the brief format used. In primary care, the use of interprofessional collaboration including both medical and psychological expertise to meet patient treatment needs is common, and such collaboration could add to the effectiveness also in general dentistry. The experiences from using this brief ACT intervention with a clinical psychologist placed at general dental clinics indicate that psychologists could aid and complement dental staff in diagnosing, treating and referring patients with various oral health issues. Moreover, clinical psychologists will enhance and strengthen the general methodological competence in research and development in clinical dentistry.

## Conclusion

In this paper, we have presented a brief behavioural ACT intervention adapted to young adult patients with dental caries. ACT has the possibility of being an effective behavioural treatment for patients with oral diseases, and interprofessional collaboration between psychologists and dental personnel opens up new possibilities to aid and treat patients with various health issues in general dental care.

## Data Availability

Not applicable as there is no raw data to provide.

## Abbreviations

ACT: Acceptance and Commitment Therapy
CBT: Cognitive Behaviour Therapy
